# Histopathological and mechanical effects of Ankaferd Blood Stopper® on wound healing in rats: an experimental model

**DOI:** 10.3906/sag-2004-177

**Published:** 2020-08-26

**Authors:** Sertaç HANCIOĞLU, Berat Dilek DEMİREL, Ünal BIÇAKCI, Seda GÜN, Ender ARITÜRK, Nursen ARITÜRK

**Affiliations:** 1 Department of Pediatric Surgery, Faculty of Medicine, Ondokuz Mayıs University, Samsun Turkey; 2 Department of Pathology, Faculty of Medicine, Ondokuz Mayıs University, Samsun Turkey; 3 Department of Ophthalmology, Faculty of Medicine, Ondokuz Mayıs University, Samsun Turkey

**Keywords:** Ankaferd Blood Stopper, wound healing, clean wound healing, burst pressure width, scar

## Abstract

**Background/aim:**

To evaluate the histopathological and mechanical effects of Ankaferd Blood Stopper (ABS) application on wound healing.

**Materials and methods:**

A total of 24 Wistar albino rats were randomly divided into three equal groups. In each group, a 3 cm-long midline vertical skin incision was performed in the back of the rats. In Group 1, the incision was sutured primarily. In Group 2, incision was left to secondary healing. In Group 3, ABS was applied to the incision. On the 10th day, burst pressure width was measured, and rats were sacrificed. The tissue samples were examined histopathologically. Statistical analysis was conducted with IBM SPSS program. P < 0.05 was considered significant.

**Results:**

The mean burst pressure widths of wound separation were 13.66 ± 0.457, 7.18 ± 2.599, and 13.66 ± 1.11 mm for Groups 1–3, respectively. The difference in burst pressure width between Groups 1 and 3 was not significant (P > 0.05) but was significant between Groups 2 and 3 (P = 0.000). The vascular proliferation median values were 1, 2, and 2, for Groups 1–3, respectively. Although the difference was significant between Groups 1 and 2 in terms of vascular proliferation score (P = 0.047), no significant difference was observed between Group 3 and others. No statistically significant difference was observed among the groups in terms of collagen score, mononuclear cell infiltration, and polymorphonuclear cell proliferation (P > 0.05). The median values of fibroblast proliferation score were 1, 2, and 3, in Groups 1–3, respectively. Fibroblast proliferation score significantly differed between Groups 1 and 3 (P = 0.003).

**Conclusion:**

ABS application results in a clean wound healing that is as strong as primary repair. However, additional studies are required to evaluate the late results of increased fibroblastic activity in the early period of ABS application alone.

## 1. Introduction

Wound healing is a progressive and dynamic event [1]. Most skin wounds heal naturally. Various agents were applied in previous studies to accelerate wound healing and increase wound strength [2–5]. Incisional wound model is often used in experimental and clinical studies. In an incisional wound model, the use of a systemic or local agent can be compared for wound tensile strength and histopathological changes in primary repair or secondary healing [6,7].

Ankaferd Blood Stopper® (ABS; Trend Teknoloji İlaç AŞ, Ankara, Turkey) is a unique Turkish medicinal herbal extract from five different plants: *Thymus vulgaris* (dried grass extract) (5.0 g/100 mL), *Glycyrrhiza glabra* (dried leaf extract) (7.0 g/100 mL), *Vitis vinifera* (dried leaf extract) (8.0 g/100 mL), *Alpinia officinarum* (dried leaf extract) (7.0 g/100 mL), and *Urtica dioica* (dried root extract) (6.0 g/100 mL) [8]. ABS has been approved for the management of external hemorrhage and dental surgery by the Ministry of Health in Turkey [9]. ABS is a promising hemostatic and possesses regenerative, antineoplastic, and antimicrobial properties against multidrug resistant bacteria [10–12]. ABS is a hemostatic agent used to control postoperative bleeding in patients with various bleeding disorders [13,14]. In addition to providing good hemostasis, ABS promotes neovascularization, which helps with rapid wound healing [5,10]. In this experimental study, we aimed to evaluate the mechanical and histopathological effects of ABS, which can be easily applied on clean surgical wounds, on wound healing.

## 2. Materials and methods

The current study was approved by the Animal Experimentation Ethics Committee of Ondokuz Mayıs University (approval no. 2017/15). All surgical procedures were performed at the Research Center for Animal Experiments at Ondokuz Mayıs University. All animals were individually caged in a room with standard environmental conditions and were fed with a standard rat diet.

### 2.1. Experimental groups and experimental model

In the study, 24 Wistar albino rats were randomly divided into three groups with 8 rats in each group. The total sample of 24 subjects, with 8 rats in each group, achieved a 92.4% power to detect differences among the means at a significance level of 0.05 [15]. Surgical incision was performed in all rats. Intraperitoneal ketamine HCl (Ketalar®, Pfizer, Turkey; 75 mg/kg) and xylazine (Rompun®, Bayer, Turkey; 0.2 mL/kg) were applied to rats to induce general anesthesia. Surgical area disinfection was provided by povidon–iodine solution after shaving the back hairs of the rats in prone position. A full-thickness 3 cm-midline skin incision was made with a scalpel on the back of the rats. Primary repair was performed in Group 1 by repairing the incision in an interrupted fashion (at 0.5 cm intervals with 5/0 silk suture), and stitches were removed on the 7th postoperative day. In Group 2, skin incision was untouched and left for secondary healing. In Group 3, only ABS was applied on the skin incision without any repair (Table 1).

**Table 1:** Designation of groups and surgical procedures.

Group	Number (n)	Experimental model
Group 1	8	Skin incision - Primary repair
Group 2	8	Skin incision - Secondary healing
Group 3	8	Skin incision - Local ABS application

### 2.2. Measurement of burst pressure width

**Figure 1:**
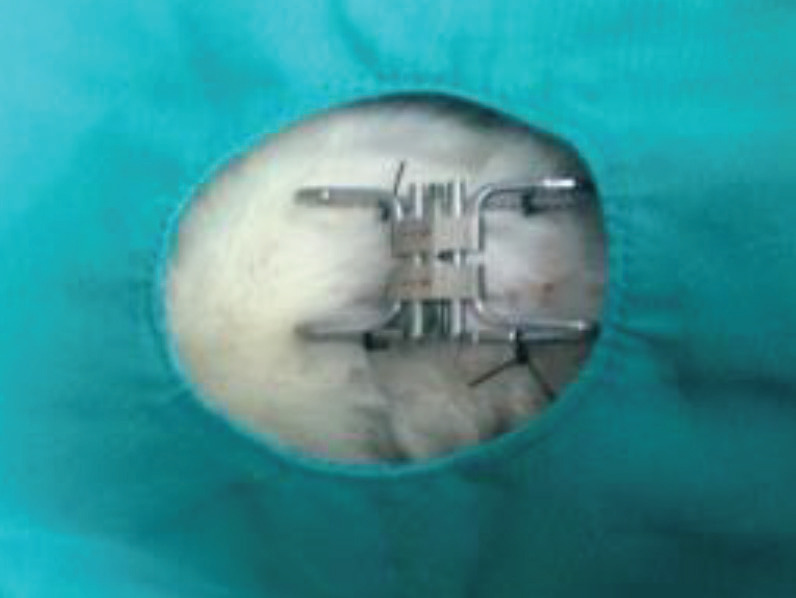
Fixation of the mechanical tension tool on the wound.

For the measurement of burst pressure width, a mechanical tension tool designed by modifying a 13 mm palatal expander was used [16]. On the 10th day after surgery, all four legs of the mechanical tensioning device were fixed to the skin at a 0.5 cm distance from both wound edges under general anesthesia in all groups (Figure 1). The mechanical tension tool was gradually opened with the help of a lever arm. The length at which the wound lips were separated was used to determine the burst pressure width (mm).

### 2.3. Histopathological examination

On the 10th postoperative day, after the measurement of wound opening, specimens including the entire incision borders were collected for histological and immunohistochemical examination.

All specimens were stained with hematoxylin–eosin (H&E), Masson’s trichrome, and reticulin stain. Tissue healing was evaluated under light microscopy. In H&Estained sections, polymorphonuclear leukocytes (PMNLs), mononuclear cells (MNLs), fibroblast proliferation, and vascular proliferation were evaluated. The parameters were scored between 0 and 3. According to this scoring system, the score was “0” when PMNLs, MNLs, fibroblast proliferation, and vascular proliferation were not observed; scores of 1, 2, and 3 indicated mild, moderate, and abundant tissue healing, respectively [17].

Collagen fibers were dyed blue with Masson’s trichrome. Collagen accumulation was scored between 0 and 3. The absence of collagen was scored as 0; accumulation of single or several collagen fibers as 1; and significant dense collagen accumulation as 3. Collagen accumulation with a score between 1 and 3 was given a score of 2 [17].

Type I collagen fibers appeared yellow, and type III fibers appeared black with reticulin dye staining. Type I/III collagen ratios of the groups were calculated [17].

### 2.4. Statistical analysis

Data were analyzed using IBM SPSS Statistics 22 (IBM Corp., Armonk, NY, USA). Descriptive statistics were expressed as mean ± standard deviation or median, depending on the distribution of variables. Categorical variables were expressed as numbers and percentages. The normality test of numerical variables was verified with the Shapiro–Wilk test. One-way ANOVA (Analysis Of Variance) with post hoc Tukey test was used for statistical analysis of burst pressure width among groups. The Kruskal–Wallis test was used for statistical analysis of histopathological parameters among groups.

## 3. Results

**Figure 2:**
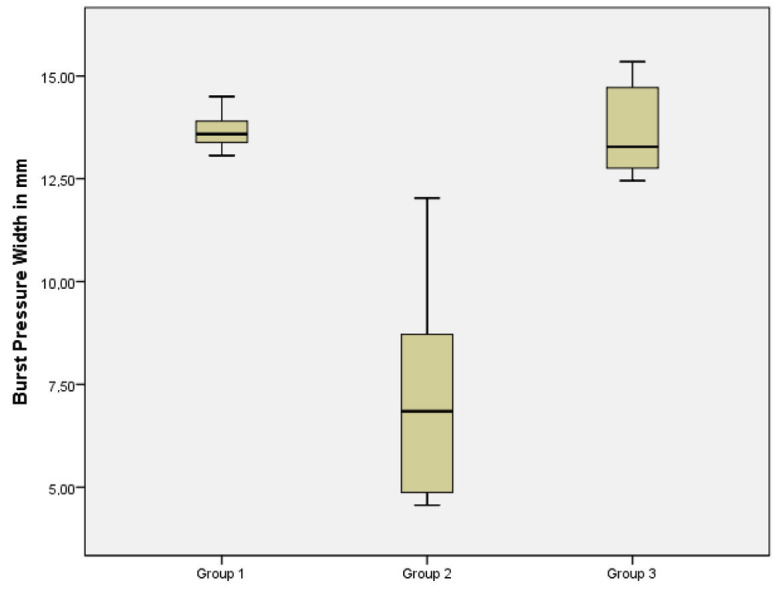
Distribution of burst pressure width (in mm) among the groups.

Normally distributed data were determined individually for all variables. Burst pressure width was normally distributed, whereas the other variables were not (Table 2). Burst pressure width was 13.66 ± 0.45 mm in Group 1, 7.18 ± 2.59 mm in Group 2, and 13.66 ± 1.11 mm in Group 3 (Table 3, Figure 2). Although the difference between Groups 1 and 3 was not significant (P > 0.05), it was significant between Group 2 and other groups (p < 0.05) (Table 3).

**Table 2:** The Shapiro–Wilk test of normality for burst pressure width, polymorphonuclear leukocytes (PMNLs), mononuclear cells (MNLs), vascular proliferation, fibroblast proliferation, and collagen scores in the groups.

	Groups	Shapiro–Wilk		
		Statistic	n	P
Burst pressure width	1	0.944	8	0.652
	2	0.916	8	0.396
	3	0.895	8	0.258
PMNLs	1	0.732	8	0.005
	2	0.641	8	0.000
	3	0.827	8	0.056
MNLs	1	0.732	8	0.005
	2	0.641	8	0.000
	3	0.798	8	0.027
Vascular proliferation	1	0.418	8	0.000
	2	0.566	8	0.000
	3	0.665	8	0.001
Fibroblast proliferation	1	0.418	8	0.000
	2	0.641	8	0.000
	3	0.724	8	0.004
Collagen score	1	0.810	8	0.037
	2	0.665	8	0.001
	3	0.798	8	0.027

P > 0.05 indicates normal distribution of the data. P ≤ 0.05 indicates abnormal distribution of the data.

**Table 3:** Distribution of burst pressure width (in mm) among the groups.

	N	Mean ± Std. Deviation	P
Group 1	8	13.66 ± 0.456^b^	0.000*
Group 2	8	7.18 ± 2.599^a^	
Group 3	8	13.66 ± 1.11^b^	

* Significant at 5% significance level; ^a,b^: For all variables with the same letter, the difference between the means is not statistically significant.

In histopathological evaluation, the median vascular proliferation score was 1 for Group 1 and 2 for the other groups. The difference was significant between Groups 1 and 2 (P = 0.047) but not significant between Group 3 and other groups (Table 4). No statistically significant difference was observed among the three groups in terms of PMNLs, MNLs, and collagen score (Table 4).

The median fibroblast proliferation scores were 1, 2, and 3 in Groups 1, 2, and 3, respectively. A statistically significant difference was observed between Groups 1 and 3 (P < 0.05). However, the difference was not significant between Group 2 and the other groups (Table 4).

**Table 4:** Comparison of median values for polymorphonuclear leukocytes (PMNLs), mononuclear cells (MNLs), vascular proliferation, fibroblast proliferation, and collagen scores among the groups (G1: Group 1; G2: Group 2; G3: Group 3).

		Min	Max	Median	P
PMNLs	G 1	0	2	1	0.121*
	G 2	1	2	2	
	G3	0	2	1	
MNLs	G 1	0	2	1	0.085*
	G 2	1	2	2	
	G 3	1	3	2	
Vascular proliferation	G 1	1	2	1a	0.047*
	G 2	1	2	2^b^	
	G 3	1	2	2^ab^	
Fibroblast proliferation	G 1	1	2	1^a^	0.003*
	G2	1	2	2^ab^	
	G 3	1	3	3^b^	
Collagen score	G 1	1	3	2	0.490*
	G 2	1	2	2	
	G 3	1	3	2	

* Significant at 5% significance level; ^a,b^: For all variables with the same letter, the difference between the means is not statistically significant.

## 4. Discussion

The skin is the body’s largest organ system, and it provides protection against microorganisms and maintains homeostasis [18,19]. The presence of microorganisms in the wound environment, the distance of wound lips, and foreign bodies delay wound healing and result in predisposition to infection [19]. Bacteremia causes morbidity and mortality through sepsis and septic shock. Wound healing is a dynamic process that involves various mediators and factors. Chemical mediators and cytokines formed after tissue damage help with hemostasis and healing [18,20]. Wound healing occurs in three phases. Owing to vascular proliferation during the inflammation phase, granulation tissue forms as the wound’s oxygenation and feeding supply increases. Fibroblasts synthesize collagen around the formed vessels and ensure the bonding of collagen with the proteoglycans they produce. The collagen produced by fibroblasts in the proliferation phase increases the contraction and tensile strength of the wound starting from day 5 and thereafter. When granulation occurs and epithelialization is completed, maturation phase starts on the 14th day. In this phase, which continues for years, fibroblasts undergo apoptosis, and the soft type III collagen is converted to a firmer type 1 collagen by fibrocytes. From the 6th week, the wound strength reaches 95% of the initial value [20].

Experimental studies evaluated the effect of different agents on wound healing [2–5,21]. These agents may be pharmaceutical/medical substances as shown by certain studies. Meanwhile, other research evaluated natural substances, similar to our study. ABS is a licensed product with a local hemostatic effect [22]. ABS is available as wound dressing, ampoule, and spray form. ABS shows its effect by forming a “protein network” in plasma and serum via erythrocytes and blood proteins, primarily fibrinogen [23]. The effect of Ankaferd starts rapidly, and the encapsulated protein network formation, which is formed by the combination of erythrocyte and blood proteins, occurs in less than a second [14]. The hemostatic, antimicrobial, antiinflammatory, regenerative, antioxidant, antiapoptotic, and antitumoral effects of ABS have been investigated in in vivo and in vitro studies [10–14,24–27]. In a study by Büyüktiryaki et al., ABS decreased the intestinal necrosis in a necrotizing enterocolitis rat model [28]. Beyazit et al. reported that ABS reduced inflammation in the developed cervicitis rat model [29]. However, no significant positive effect of ABS was observed on sciatic nerve injury and tendon healing [30,31].

Akalın et al. examined the full-thickness skin defect on the back of rats in three groups. They examined the histopathological changes and wound contraction percentages in the ABS, secondary healing, and wound dressing groups. The ABS group showed statistically better results than the other groups [7]. In our study, we compared the primary repair and secondary healing of full-thickness skin incision in rats with burst pressure width and histopathological evaluation.

A study claimed that the *Glycyrrhiza glabra* content of ABS decreases vascular proliferation, whereas another research reported that ABS increases vascular proliferation [32,33]. However, we could not demonstrate any significant positive nor negative effect of ABS on vascular proliferation. As the absence of negative effect on vascular proliferation could be demonstrated, deleterious effects on wound healing are unlikely. In our study, no significant effect of ABS on MNLs and PMNLs was observed. Verhofstad et al. showed that increased macrophage level causes poor wound healing [34]. Another study showed that local ABS decreased the macrophage level after sleeve gastrectomy in rats [35].

Boran et al. showed that ABS increased fibroblasts in the wound area with chemotactic activity [12]. In our study, the three-fold increase in fibroblasts in the ABS group relative to that in the primary repair group is parallel with findings in the literature. The fibroblast-promoting effect of ABS may be due to its antiapoptotic properties. The inhibition of the apoptosis of fibroblasts during the maturation phase with activation of fibrocytes may lead to increased and irregular collagen production in the following period. In our study, no significant difference was observed in the scores on collagen, which is product of fibroblasts, among the groups. Given that this study was terminated in the proliferation phase, further complementary research is required to address the risk of hypertrophic scar development

Cancan et al. showed that ABS increased the mechanical strength of anastomosis in the rat colon [33]. In our study, the burst pressure width was standardized and measured on the 10th day after the surgical procedure. The burst pressure width of the ABS group was similar to that of the primary repair group, in which the wound lips were repaired. The burst pressure width of the ABS group was significantly higher than that in the secondary healing group, in which the wound was left for secondary healing. Primary repair provides the best wound healing effect to the best of our current knowledge [36]. The effect of ABS in providing mechanical strength that is as strong as primary repair can be attributed to the increased fibroblast activity.

Beside its hemostatic effect, ABS promotes wound healing. In our study, the ABS group exhibited a contraction force as strong as that in the primary repair. However, studies with more groups and dependent variables are needed for clearer interpretation of the histopathological features. We believe that the effects of ABS on wound tensile strength, collagen production and transformation, and hypertrophic scar development due to increased fibroblast activity in the maturation phase can be evaluated more in detail. We think that our study will become a cornerstone for future experimental studies.
